# A qualitative examination on the implementation of participatory “A”rt-based activity on “Health” of older community-dwellers: what worked for the Singapore A-Health Intervention?

**DOI:** 10.3389/fmed.2023.1238563

**Published:** 2023-12-19

**Authors:** Stephanie Hilary Xinyi Ma, Michael Koon Boon Tan, Shannon Shuet Ning Goh, Gabriellia Yeo, Alicia Teng, Yilin Yang, Kévin Galéry, Olivier Beauchet, Andy Hau Yan Ho

**Affiliations:** ^1^Action Research for Community Health (ARCH) Laboratory, Psychology Program, School of Social Sciences, Nanyang Technological University, Singapore, Singapore; ^2^Lab4Living, Culture and Creativity Research Institute, Sheffield Hallam University, Sheffield, United Kingdom; ^3^National Gallery Singapore, Community and Access, Singapore, Singapore; ^4^Research Centre of the Geriatric University Institute of Montreal, Montreal, QC, Canada; ^5^Departments of Medicine and Geriatrics, Faculty of Medicine, University of Montreal, Montreal, QC, Canada; ^6^Lee Kong Chian School of Medicine, Nanyang Technological University, Singapore, Singapore; ^7^Palliative Care Centre for Excellence in Research and Education, Singapore, Singapore

**Keywords:** participatory arts, museum, social prescribing, wellbeing, older adults, implementation science, qualitative inquiry a-health experience, Singapore

## Abstract

**Introduction:**

Art and museum-based interventions are gaining increasing recognition for their potential as low-risk activities for older adults, offering numerous physical, cognitive, and emotional benefits. However, there remains a dearth of knowledge regarding the science of implementation as well as the factors and processes that contribute to their effectiveness from the perspectives of intervention participants.

**Methods:**

The current research draws on the qualitative evaluation data obtained from a larger mixed-method randomized control trial that evaluated a standardized Participatory “A”rt-Based Activity On “Health” of Older Community-Dwellers—the Singapore A-Health Intervention. Adopting a participatory action research approach, the primary objective is to critically examine the lived experiences and health impact of the Singapore A-Health Intervention with a secondary objective to uncover strategies for optimized implementation outcomes. All 56 participants who completed the intervention filled out a program evaluation survey and a nested sample of 30 participants completed a series of acceptability focus groups.

**Results:**

Descriptive analyses of the program evaluation survey data revealed that 96.2% of participants were satisfied with the overall experience of the Singapore A-Health intervention (*M* = 9.00, SD = 1.76), reported that the intervention positively impacted their quality of life (*M* = 8.90, *SD* = 1.43), and social wellbeing (*M* = 8.92, *SD* = 1.43). Thematic analysis with a grounded theory approach on the qualitative focus group data revealed three interrelated themes detailing how the Singapore A-Health Intervention contributed to positive health and wellbeing outcomes (1. A-Health Experience, 2. Wellbeing Outcomes, 3. Enabling Factors) and nine subthemes (1a. Intellectual Stimulation, 1b. Positive Stress, 1c. Peer Interaction, 2a. Interpersonal Bonds, 2b. Personal Growth, 2c. Mindful Living, 3a. Integrated Support, 3b. Session Design, 3c. Mode of Engagement).

**Discussion:**

This investigation provides important insights to the Singapore A-Health intervention’s effectiveness for enhancing wellbeing among older adults, as well as the factors that enable successful program implementation. These findings offer a culturally unique perspective on the benefits of art and museum interventions, while underscoring the imperative need for strong partnership and collaborations among community stakeholders in supporting the health and wellbeing of ageing populations.

## 1 Introduction

### 1.1 Background

The global population is ageing at an unprecedented rate and it is associated with declining physical, cognitive, social, and psychological health (1). As the ageing population continues to grow, there will be an inevitable increase in the demand for healthcare services. Consequently, there is an urgent need to devise innovative approaches to alleviate the burden on the healthcare system. The field of art and health has gained increasing recognition for its potential to promote holistic health across the lifespan and it has been found to be a safe activity for older adults, offering numerous physical, cognitive, and emotional benefits (2–5). Participation in art programs also provide opportunities for social interaction and connectedness, which are crucial for combating social isolation among older adults (6). Although there is a growing amount of evidence supporting the health promoting benefits of art engagement for older adults, most studies have predominantly focused on Western populations, with limited exploration of the experiences of older adults from diverse ethnic backgrounds.

Moreover, many studies investigate the abundant benefits of art participation, but there remains a dearth of knowledge regarding the factors that contribute to their effectiveness from the perspectives of the participants and service providers. An implementation science approach could significantly enrich the investigation of arts programs by providing a comprehensive framework to investigate effective program delivery and outcomes (7). This approach was adopted in various fields to improve patient care quality and service delivery (8–10), and is particularly useful for the evaluation of art-based interventions where the context varies across settings and is greatly shaped by cultural influences. An implementation science approach could also aid the development of guidelines and recommendations for the continued delivery of art programs beyond the research study (11). By examining factors that influence successful program implementation, the gap between research evidence and practice could be bridged with this approach (12).

In 2015, a 12-week art and museum-based intervention for health (i.e., A-Health) was developed through a collaborative effort between the Centre of Excellence on Longevity of McGill University Canada, together with the Montreal Museum of Fine Arts. The preliminary findings supported the effectiveness of the 12-week program in improving mental and physical health (13, 14) which led to the empirical expansion of the A-Health study through an international Randomized Control Trial (RCT). The original 12-week A-Health framework was adapted for the Singaporean context (i.e., Singapore A-Health Intervention) with culturally specific modification informed by a participatory action research approach (15). It was developed in partnership between the Action Research for Community Health (ARCH) Laboratory at Nanyang Technological University (NTU), Lab4Living at Sheffield Hallam University, and the National Gallery Singapore (the Gallery), a renowned visual arts institution with the largest public collection of Singapore and Southeast Asian modern art. Results from the mixed-methods randomized control trial (RCT) of the Singapore A-Health Intervention (reported elsewhere in this journal) (See 10.3389/fmed.2023.1238562) provided further evidence to support the efficacy of standardized art and museum-based interventions for improving the physical and mental health of community-dwelling older adults. Drawing on the qualitative evaluation data of the mixed-methods RCT and adopting a participatory action research approach, this study aims to critically examine the lived experiences and health impact of the Singapore A-Health Intervention on participants who have completed the program, with a secondary objective of uncovering strategies for optimized implementation outcomes.

## 2 Methodology

### 2.1 Research design

Qualitative methods were utilized to address the research goals due to its ability to address complex questions and uncover intricate dynamics in the implementation processes (16). This research was shaped by a constructivist paradigm (17, 18), guided by a relativist ontological stance and a constructionist epistemological stance (19) to understand experiences of the Singapore A-Health Intervention through the perspective of the research participants.

### 2.2 Sampling

Participants recruited for the RCT were community dwelling older adults above the age of 60, fluent in English, and had internet access to complete online questionnaires. Individuals who were unable to provide informed consent or formally diagnosed with mental health conditions such as cognitive impairments or major depressive disorders were excluded from the study. Participants were recruited through the gallery, social media platforms, and partnering eldercare agencies in Singapore. For the qualitative evaluation, eligible participants were those assigned to the intervention group and attended at least 80% of the sessions.

### 2.3 Study procedures

The study was implemented in two phases: a pilot study to refine the intervention protocol in March 2021, and a full study to evaluate the refined intervention protocol in September 2021. Participants referred by the eldercare centers or who registered their interest in the study online form were contacted by a member of the research team via audio or video call. During the call, participants were given the opportunity to ask questions about the study before completing the informed consent form. Thereafter, they were sent a personalized link to an online baseline assessment and allocation outcomes were then revealed to participants upon successful completion of the baseline assessment. Intervention group participants were assigned to the 12-week A-Health Singapore intervention held at the gallery while the control group were not offered any art or museum-based activities. During the intervention period, participants were invited to complete four standardized physical and psychological health assessments and received SGD$80 for completing all four self-administered questionnaires. The quantitative findings are reported elsewhere in this journal.

Four acceptability focus group discussions and a program evaluation survey were conducted with intervention group participants after the 12-week program to evaluate program and implementation effectiveness. The aim of the focus group was to gather in-depth insights and perceptions of the participants regarding their experiences of the A-Health Singapore program. A semi-structured topic guide was developed, covering topics such as the (a) overall experiences of the 12-week A-Health Singapore program, (b) perceived health and psychological impact of the program, (c) motivating and inhibiting factors of engaging in the program, and (d) an evaluation of the intervention framework and implementation processes. The focus groups were conducted online via Zoom by a research staff and a trained co-facilitator, and the duration of the discussions were between 60 and 90 min. The discussions were audio-recorded and transcribed using intelligent verbatim transcription for data analysis. The evaluation survey assessed various areas of study implementation including recruitment, facilitation, session content, and perceived impact of the intervention. Participants were invited to rate their agreement on a series of statements using a Likert scale (e.g., I am satisfied with the overall experience of the A-Health Singapore research study, where 1 = strongly disagree and 10 = strongly agree). An open-ended section was included at the end of the survey for participants to express their opinions on topics not covered in the Likert scales. Confidentiality of the participants was ensured by assigning participants with a unique ID and the omission of all identifying information in the transcripts. Ethnographic notes of *ad hoc* group discussions with gallery docents and intervention facilitators were also documented to illuminate the interventionist and developer perspectives; these notes served to inform data analysis and data interpretation.

### 2.4 Pandemic-influenced procedures

Due to the COVID-19 pandemic, the implementation of the study was impacted by nationwide health and safety regulations to prevent the spread of the virus. Firstly, group sizes were reduced, and participants were grouped into sub-groups of three to eight during the intervention. They were also required to maintain a physical distance of at least one meter and were not allowed to interact with participants from other subgroups. Secondly, some sessions were run online due to the lockdowns implemented in Singapore (20). Specifically, online sessions were conducted on the twelfth week of the pilot study, and fourth to eighth week of the full study. Thirdly, the enforcement of vaccination differentiated measures restricted access to the gallery for those who were unvaccinated (21). This resulted in the implementation of a hybrid format where vaccinated individuals attended the onsite sessions, while unvaccinated individuals engaged in the program online. The hybrid sessions were conducted on the ninth to twelfth week of the sessions in the full study.

In response to the challenges posed by the COVID-19 pandemic, a comprehensive online contingency plan was developed to ensure a seamless transition from onsite to online programming. The program adapted the curriculum to suit the online format, incorporating interactive elements such as live-streamed gallery tours, online art demonstrations, and facilitated discussions via a secured video-conferencing platform, Zoom. Several measures have been put in place to ensure smooth transition and maintain the quality of the intervention. Firstly, the research team provided step-by-step guidance through the creation of user manuals and video tutorials to help participants navigate the online platform and access the virtual sessions comfortably. Secondly, regular communication channels through a dedicated WhatsApp group have been established to address participants’ questions promptly. Thirdly, the facilitators ensured that the online sessions were conducted at a manageable pace that allows participants to grasp new art techniques and engage in meaningful discussion in an interactive virtual learning environment. This was achieved by including breakout groups for personalized feedback by the artist and co-facilitators, and to encourage interaction among participants.

A hybrid version was developed to accommodate the vaccination differentiated measures and ensure inclusivity in the program. Vaccinated individuals attended the sessions onsite, while those who were unvaccinated attended the sessions online. Measures were taken to effectively engage all participants. The artist provided in-person guidance for the onsite participants, and simultaneously ensured that online participants were actively involved by providing instructions and offering feedback on their artwork. To ensure that online participants received the same level of attention and support as their onsite counterparts, a dedicated research team member was assigned to facilitate the video call and promptly address any queries or technical issues raised by online participants. For the final session of the program in a hybrid format where participants showcased their final artworks and engaged in group sharing, the artist created a collaborative environment where both onsite and online participants could interact. The dedicated research team member projected the video conference onsite for online participants to present their artworks and express their inspiration. The artist then invited onsite participants to ask questions to their peers and online participants were also provided with the opportunity to share their thoughts on the onsite exhibitions, encouraging discussion across platforms.

### 2.5 Intervention design

**FIGURE 1:**
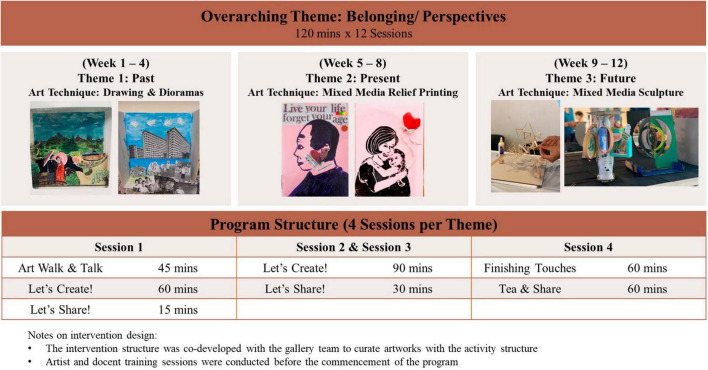
Singapore A-health intervention structure.

The Singapore A-Health Intervention spanned 12 weeks, with each weekly session lasting for 2 h. The structure of the program adhered to the original A-Health framework, and the topic and activities were jointly developed with the gallery staff, docents, artists, and research participants using a participatory action research approach (15) for cultural specificity. The primary goal of the program was to provide participants with fundamental art appreciation skills and art techniques through active engagement with the museum collection. The program was structured around three thematic domains of the past, present, and future. In each thematic domain consisting of 4 weeks, participants explored the themes and learnt relevant art techniques such as mixed media relief printing or cardboard sculpturing. Participants were tasked with creating an artwork related to the theme and incorporating the learned techniques. The structure of each thematic domain followed the same structure: the first week involved a 45-min docent-led gallery tour on three selected pieces of artworks, followed by a 75-min artist-led brainstorming session where participants were introduced to the techniques and discuss ideas on the artwork to be created. The subsequent three sessions involved further guidance from the artist and a scaffolded delivery of art techniques for participants to incorporate their learning to their artwork. At the end of each domain, there was a showcase where participants presented their artwork, shared their artistic journey, and engaged in meaningful conversations about the themes explored. The intervention outline and sample artworks can be found in Figure 1.

### 2.6 Data analysis

The responses collected from the program evaluation survey was analyzed using descriptive statistics. The focus group discussions were analyzed using thematic analysis with a grounded theory approach (22) to provide insights to the participant’s experience and impact of the Singapore A-Health Intervention. Data familiarization followed by line-by-line coding of all verbatim responses from the focus group discussions were first conducted. Codes were then clustered into themes and subthemes, and axial coding was subsequently performed to develop conceptual theme categories. Selective coding was then utilized to illuminate the relationship between theme categories. Emergent themes and subthemes were refined by multiple reviews with appointed members of the research team and were presented to the larger research team for refinement and revision. Major theme categories and themes were defined, operationalized, and mapped into a proposed conceptual framework. Research rigor was maintained via a multi-pronged strategy including documentation in an audit trail, prolonged engagement with the research data, regular team meetings and peer debriefing. Moreover, the analysis was enriched with data triangulation from the ethnographic notes of *ad hoc* docent and facilitator group discussions, as well as investigator and theory triangulation.

## 3 Results

### 3.1 Participant demographics

A total of 56 participants were randomized into the intervention group and 2 participants dropped out during the program due to ill health and work commitments. Intervention group participants were between the age of 60 and 80 years (*M* = 65.8, SD = 3.40), with a female majority (73.2%) and of Chinese ethnicity (96.4%). Participants attended an average of 11 out of 12 sessions. For the focus group discussions, a nested sample of 30 participants from the intervention group were invited to participate. The average age of this group was 66.2 years (SD = 2.93), majority were females (70%) and of Chinese ethnicity (96.7%). They were mostly retirees (70%) and had a bachelor’s degree (36.7%). These participants were also physically healthy (60%) and physically active (100%). For more information on the participant’s demographics, please refer to Table 1.

**TABLE 1:** Baseline demographic information.

	Intervention (*n* = 56)	Focus group (*n* = 31)
Age (year, mean ± SD)	65.8 (3.40)	66.2 (2.93)
**Sex (n, %)**		
Male	15 (26.8)	9 (30.0)
Female	41 (73.2)	21 (70.0)
**Marital status (n, %)**		
Single	12 (21.4)	7 (23.3)
Married	37 (66.1)	22 (73.3)
Divorced/separated	4 (7.1)	
Widowed	3 (5.4)	1 (3.3)
**Highest education attained (n, %)**		
GCE “N,” “O” level, GCE “A” level or ITE/higher Nitec and below	16 (28.6)	9 (30.0)
Polytechnic diploma or professional certificate	9 (16.0)	6 (20.0)
Bachelor’s degree	24 (42.9)	11 (36.7)
Postgraduate degree	7 (12.5)	4 (13.3)
**Ethnicity (n, %)**		
Chinese	54 (96.4)	29 (96.7)
Indian	1 (1.8)	-
Other: Biracial	1 (1.8)	1 (3.3)
**Employment status (n, %)**		
Full-time employed	6 (10.7)	2 (6.7)
Part-time employed	14 (25)	7 (23.3)
Unemployed/retired	36 (64.3)	21 (70.0)
**Presence of chronic illness (n, %)**		
Yes	25 (44.6)	12 (40.0)
No	31 (55.4)	18 (60.0)
**Practice of physical activity (n, %)^a^**		
Yes	56 (100)	30 (100)
No	–	–
Number of A-health sessions attended (Mean ± SD)	11.3 (1.73)	11.6 (0.62)

^*a*^Regular physical activity (walking, bicycle, etc.) at least 1 h per week in the past month.

### 3.2 Program evaluation survey findings

A total of 53 participants completed the post-intervention participant satisfaction survey. Descriptive analyses revealed that 96.2% (*M* = 9.00, SD = 1.76) were satisfied with the overall experience of the Singapore A-Health Intervention. A total of 96.2% of the participants agreed that engaging in the activities positively impacted their quality of life (*M* = 8.90, SD = 1.43) and social wellbeing (*M* = 8.92, SD = 1.43). A total of 98.1% (*M* = 9.06, SD = 1.36) agreed that their physical health was positively influenced by the program. In terms of program implementation, 96.2% were satisfied with the overall gallery tours (*M* = 8.58, SD = 1.73) and the art making workshops (*M* = 9.20, SD = 1.40), and 98.1% were satisfied with the support and guidance received by the facilitators (*M* = 9.30, SD = 1.20).

### 3.3 Acceptability focus group discussion findings

**FIGURE 2:**
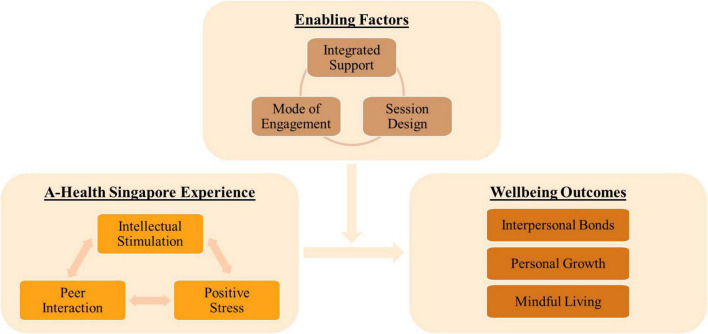
Qualitative findings of the Singapore A-health intervention study.

Four groups were conducted with 30 participants from the first and second phases of the study. Thematic analyses with a grounded theory approach revealed three interconnected themes and nine subthemes (see Figure 2). The first theme illustrated the *A-Health Singapore Experience* which included the subthemes of *intellectual stimulation, positive stress*, and *peer interaction*. The second theme demonstrated the perceived *wellbeing outcomes* stemming from the participant’s engagement in the 12-week program which include the subthemes of *personal growth*, *mindful living*, and *interpersonal bonds*. The final theme illuminates the *enabling factors* which appeared to moderate the relationship between the A-Health Singapore experience and wellbeing outcomes. The subthemes include *integrated support*, *mode of engagement* and the *session design*.

#### 3.3.1 Theme 1: A-Health Singapore experience (*n* = 24; shared by 24 out of 30 participants)

The participants recounted their experience of the 12-week program and described how they were intellectually stimulated by the unique program contents. They found the session contents and project deliverables challenging, which elicited positive stress. Participants shared various strategies to cope with the stress including seeking support from their peers and self-learning after the weekly sessions. While some participants were empowered by the peer interaction, others felt internally pressured due to personal comparisons with their peer’s progress which further contributed to their stress levels. Although participants reported feeling stressed during the program, they concurred that it was a fulfilling experience to complete the program.

##### 3.3.1.1 Subtheme 1a: intellectual stimulation (*n* = 16)

The Singapore A-Health Intervention were deemed to be educational, and it challenged participants to think creatively and explore various perspectives on art. A participant shared that “*it challenges my mind to think outside the box. Most of the time I think that art is just drawing, painting, or coloring within the boundaries but this project gave me a better perspective on art, viewing art from different angles, different way of looking at things, and then broadening my mind (AHS081, 65-year-old, female).”* These challenges were also perceived as an avenue for problem-solving which motivated participants to learn outside of the sessions. As explained by a participant, “*because it was my first time working on the three projects, I had to do a lot of research*… *I was on YouTube all the time*… *with every little thing [referring to the information found], I expanded my search (AHS061, 65-year-old, female).”* Their motivation to learn was also noticed by a docent where she learnt that the participants “*stayed up late into the night to do google searches or watch YouTube to come up with ideas and complete their artworks (Docent, NGS01)*.” Furthermore, the process of conceptualizing the art pieces was engaging and stimulating for the participants, as mentioned by this participant, “*[the project] keeps my mind wondering whether I should change [my original plan for the art piece]. I changed my ideas along the way, but stuck to my original plan*… *I now understand that there is a conceptualization process behind the art. I have learnt a lot and I’m happy about it (AHS101, 64-year-old, female).”*

##### 3.3.1.2 Subtheme 1b: positive stress (*n* = 7)

Many participants entered the program with a belief that they had no artistic talents, and this was a source of stress during the process. A participant revealed that “*it was a love-hate relationship [with the program]. I loved it, but I felt so stressed*… *because I was not good at art. But after going through all the sessions, I felt amazing that I went through it. I recalled my son telling me*… *that he had never seen me work so hard, dismantling the sculpture and trying again. This made me feel amazing as I was able to sit down and complete all three art projects* (*AHS056, 71-year-old, female).”* Another source of stress was the uncertainty stemming from the art process where they were tasked to create an art piece related to the themes and skills taught but were not provided with a reference piece. This participant explained that “*I was initially quite excited, but I felt stressed because I believed that there was a certain expectation of us to have some background [in the arts]*… *it felt pressurizing to do something that I did not know. Although it seemed like an easy topic of creating a sculpture of anything I liked, it was hard to think of how I could use cardboard to create the sculptures (AHS023, 65-year-old, female).”* In addition, although the facilitators reminded participants that the intent of the program was to explore and have fun, many participants were still highly competitive and motivated to excel which exacerbated the stress. One participant narrated that “*I felt lost at the second piece [mixed media relief printing] and went to the sink to rinse and redo my art piece multiple times. It got worse and at last, I stopped. I was not satisfied with my work, but the facilitator assured me that the artwork did not have to be perfect, and as long as the work comes from the heart, it was good enough (AHS044, 71-year-old, female).”* A facilitator of the program also reflected that “*some participants were unsure of themselves or lack confidence*… *[while others] embraced the attitude to try their best and encourage their fellow teammates (Researcher and co-facilitator, FAC01)*.”

##### 3.3.1.3 Subtheme 1c: peer interaction (*n* = 13)

The Singapore A-Health Intervention was a platform to meet others with similar interests, where “*having a group like that gives seniors a chance to interact and enjoy some form of socializing and to learn what others do (AHS023, 65-year-old, female)*.” This was also supported by an observation made by a gallery docent where she found that some of her participants “*were lonely and they wanted in-person interaction and meet other people through the program*… *and the program was a positive way for them to come out and meet other people, to learn something new, and have a creative output (Docent, NGS05)*.” A supportive culture jointly created by the facilitators and participants of the program nurtured inspiration and promoted healthy competition potentially alleviating the stress the participants experienced. A participant illustrated how his classmates were both a source of stress and support to him as “*I was most stressed by all the top students in my team. Overall, I enjoyed the teamwork in this program. It was a very nice thing to mix around with people and get inspired to do the best I could where we built on one another’s strengths (AHS090, 63-year-old, male)*.” Furthermore, the shared experience of navigating through the stress and challenge of the program also encouraged interaction as illustrated by this participant, “*by articulating my frustration to a friend and then finding out that she shared the same frustration helped a lot as we talked and gave each other the courage to try something. It was good to have somebody with me in a situation where we were doing new things (AHS020, 67-year-old, female).”*

#### 3.3.2 Theme 2: wellbeing outcomes (*n* = 22)

Although quality of life was not statistically significant in the quantitative findings, the participants provided rich and descriptive narratives of experiencing positive outcomes in several domains of wellbeing including interpersonal bonds, personal growth, and mindful living.

##### 3.3.2.1 Subtheme 2a: interpersonal bonds (*n* = 10)

The 12-week Singapore A-Health Intervention offered opportunities for interaction and relationship building which resulted in social connectedness with fellow attendees. For example, a participant shared that “*the part that surprised me was how well we could get along because we didn’t know each other [prior to the start of the program]. As the 12 weeks went by, everyone got more and more friendly, and that meant a lot to me (AHS038, 69-year-old, female).”* Another participant reflected that even though 12-weeks was a short period of time, the relationships formed were akin to longtime friends, “*for this short-term project, I would not have expected that we could so nicely bond with each other. I was so happy and comfortable that I could just joke. I felt like we had known each other for a long time*… *it was not just the physical artwork, but the people in the group that mattered a lot more for me to fully benefit from this research project thoroughly and in a wholesome way (AHS003, 63-year-old, female).”* Participants also shared their experiences with their family and friends, and this strengthened their interpersonal relationships with others outside of the program. For example, a participant who did not usually appreciate art raised the topic at home and “*it was funny, and everyone had a good laugh. The experience was quite good as the whole session lifted the atmosphere and added harmony to family gatherings. Sharing my experience with my friends was quite fun too (AHS127, 66-year-old, male)*.” Facilitators of the program also observed a sense of camaraderie among participants where “*the artmaking process helped them form bonds and develop a sense of social involvement and belonging to a group. From the ideation to the creative process, there were much brainstorming through discussions, opinions, sharing ideas, and getting help*… *these interactions may create a new sense of identity for them to feel meaningful*… *which can contribute to better wellbeing (Researcher and co-facilitator, FAC05)*.”

##### 3.3.2.2 Subtheme 2b: personal growth (*n* = 12)

Participants reported learning new skills and experience a sense of satisfaction from completing the Singapore A-Health Intervention. A participant “*thought it was quite nice to do something different and learn something new. It’s in this course that I challenged myself to learn new things, and it was a wonderful experience (AHS023, 65-year-old, female).”* The complexity of the program may have elicited stress among the attendees, but it was an opportunity for growth where this participant likened the experience to the joys of childbirth, “*I thought that the challenge was good because I discovered that I was averse to doing anything new*…*and because [creating the art pieces as deliverables] was built into the workshop, I had to do something [despite her resistance to trying something new]. I agonized over weeks, days, and nights, and at the end of it,” “gave birth” to something [referring to her artwork]. It was like labor pains, or a crescendo to a piece of work. When I “birthed” the artwork, I felt so happy. I felt fulfilled that I managed to do something different, managed to go beyond what I would normally would do, something different from my normal ability (AHS020, 67-year-old, female).* Participants also gained a perspective change regarding their views of their ability. A participant initially felt that “*I couldn’t do the art pieces but made up my mind to try with the encouragement from the session facilitators.”* She later had a realization that by being open to experiences, she will “*know how far I can go; and if I did not try, I will forever feel that I cannot do it*.” As a result of her openness of mind and resilience, she “*saw the results and really enjoyed the course (AHS056, 71-year-old, female).”* Program facilitators also witnessed many participants being more open to experience as the program progressed and explained that “*the sense of achievement that they get from creating their art piece and compliments from others also surmounts personal barriers because a lot of participants come in and voice their fears of not being good at art or were confused with the themes. But to be able to materialize something, see that they can create something, and have the physical representation of their achievement makes them very proud. It’s a reminder of what they can achieve despite their doubts (Researcher and co-facilitator, FAC05)*.”

##### 3.3.2.3 Subtheme 2c: mindful living (*n* = 10)

The Singapore A-Health Intervention inspired some participants to be more mindful in their everyday lives and curious of their surroundings. A participant described that the program “*increased my observation of my surroundings. When I went to Gardens by the Bay (a nature park in Singapore) and stopped to look at some of sculptures, I tried to see it from a different point of view. So, (the A-Health Singapore intervention) gave me a better sense of perspective of my surroundings (AHS081, 65-year-old, female).”* Moreover, another workshop attendee added that “*I started to notice that the trees have many different shades of green, and how the colors of the tree leaves have changed, which I never really noticed before. I also became more aware of nature and think that I’m starting to appreciate art (AHS079, 68-year-old, female).”* Another facet of mindful living is the awareness of an individual’s thoughts and emotions. The program created opportunities for reflection and self-expression which brought solace during the COVID-19 pandemic. This participant disclosed that “*I was very impacted [by the pandemic] in so many ways and I just couldn’t run away from it when I’m doing the art pieces*… *The abstract art activity gave me an opportunity to convey the things that have been impacting my life.”* Eventually, “*I couldn’t imagine that I was able to use something like that to articulate and express what I was feeling inside. That peace gave me the most satisfaction (AHS020, 67-year-old, female).”* Facilitators also witnessed the participants reveling in the present moment by “*getting into a state of flow where they really lose track of time when they are doing their art pieces*… *it is a beautiful thing to see the participants giving their all and being very serious about this. The benefits of this flow are heightened concentration and all sorts of things associated with flow (Researcher and Co-facilitator, FAC05)*.”

#### 3.3.3 Theme 3: enabling factors (*n* = 28)

This theme covers useful elements to optimize the running of the Singapore A-Health Intervention. Respondents highlighted the importance of having effective communication from the session facilitators, a clear and structured curriculum, as well as the pros and cons of the in-person, hybrid, and online sessions during the pandemic. This discussion was corroborated with inputs from the session facilitators. Taken together, these factors influenced the overall experience of the program and supported positive wellbeing outcomes.

##### 3.3.3.1 Subtheme 3a: integrated support (*n* = 20)

Implementation of the Singapore A-Health Intervention involved multiple facilitators: a docent for the guided tours, an artist for the art activities, and a research officer who co-facilitated the sessions and liaised with the participants. Each facilitator played a unique role in shaping the participant’s experience, while contributing to the same goal of improving the participant’s wellbeing through the program. For instance, the docents helped participants learn “*how to appreciate the different perspectives*… *and helped made sense of the painting as I was not familiar with some of the artists, so giving a background of the artist’s frame and perspectives adds understanding to what was being drawn (AHS109, 64-year-old, female).”* The facilitator and researchers assisted the participant as they navigated the themes and program content, where their “*frame of mind was framed by the artist’s input, and it actually helped a lot. Many would have attended the session without thinking in-depth about what the art pieces were about and what we were supposed to do (AHS127, 66-year-old, male).”* With different facilitators in the program, it was important for facilitators to provide cohesive instructions to avoid confusion. A participant shared that “*a number of them did not come from an art background, so the terms and concepts were quite new. The tours could be more explicit to inform participants about the concept of their artworks*… *and how the museum tours were linked (AHS076, 70-year-old, male).”* Participants also expressed the need for clear and empathetic communication to engage the group and manage program expectations.

This was also echoed in the *ad hoc* group discussions with the Singapore A-Health Intervention facilitators, where they concurred that facilitation skills such as active listening, the use of probing questions, and the ability to moderate discussions with diverse viewpoints were useful for facilitating interactive art and museum program such as the Singapore A-Health Intervention tours. As this facilitator succinctly put, “*there is the art of questioning, the art of listening, the art of validation, and quick thinking on your feet to draw and tie everything together so that everybody feels validated and contributed to the conversation (Docent, NGS06).”* In terms of program execution, a strong collaboration between stakeholders was key to effective implementation. For example, the enforcement of lockdowns in Singapore required swift transition from an onsite to online program which required many changes, the program organizer shared that, “*if I had to do this by myself, this whole thing would definitely crash and burn, but the team is great and there has been a lot of communication between the parties running the study because everyone was very open to any last minute changes, and they were very accepting which was definitely very helpful (Researcher and co-facilitator, FAC01)*.” Moreover, a supportive network of facilitators was essential for successful sessions. The docents were forthcoming with sharing resources and ideas with each other to enhance the participant’s experiences of the tour, while the artists and co-facilitators met before sessions to discuss session plans. This strong support system motivated facilitators to provide quality sessions to the participants and improve service delivery collectively.

##### 3.3.3.2 Subtheme 3b: session design (*n* = 19)

The Singapore A-Health Intervention consisted of museum tours and artmaking activities that explored the themes of the past, the present, and the future. For each theme, participants used different techniques taught by the artist to create an art piece. Participants appreciated the activities and found the session flow appropriate. This participant explained the link between activities, “*the tour and artmaking activities were interlinked. If I were given the tour only, then I would be touring and going back [home]. There was nothing to take back. But now with the tour and art activity, I was made to think and create (an art piece) and have something that comes out of that (The tours and art activities) must go hand-in-hand (AHS061, 65-year-old, female).”* This point was also highlighted by the facilitators, who observed that the scaffolding incorporated into the framework allowed easy entry for participants with little experience with art. Despite this, some participants had difficulties realizing the theme in their artworks due to challenges connecting the themes. The fixation on themes stifled their creativity and was a source of stress for some participants. For example, one participant “*was locked into thinking that the first project should be about the past”* but wondered “*how far in the past was considered as the past and had a lot of liberty looking into it”* and suggested that “*it didn’t need to be (related to) the past, present or future. I thought that the project should give more freedom to me (AHS090, 63-year-old, male).”* Other participants opined that the structure is necessary to kickstart the creative process, “*if there weren’t the themes of past, present, and future, it would then be free for everybody and too unrestricted. In the first tour about the past of Singapore, many participants talked about their past experiences and this inspired me to create the old market scene in my diorama (AHS130, 67-year-old, male).”* This finding suggests that while the flow and art activities were appropriate, the intervention protocol was not a one-size-fit-all solution and adjustments could be made to the delivery of the program to cater to the unique learning needs of the older adults.

A standardized intervention design also provided structure and alignment across facilitators, which was important for a program involving multiple facilitators. The lead artist highlighted that “*the structure provided easier communication among all who were involved (Artist-facilitator, FAC04)*,” at the same time allowing flexibility for the facilitators to “*bring their own passion, own ways to share something, and their enthusiasm (Docent, NGS05)”* which in turn positively influenced the sessions. Additionally, having a protocol enabled better resource allocation from a logistical standpoint and more bandwidth on the facilitator’s end to focus on improving participant experiences. For example, the research coordinator of the study explained that “*execution was easy because there was a clear plan of action*… *and it was very easy to know what to look out for and how to support the program in terms of the tour, the artmaking, and the preparation for the participants. During the setup, I knew exactly what materials to prepare and how to make things easy for the participants (Researcher and co-facilitator, FAC02).”*

##### 3.3.3.3 Subtheme 3c: mode of engagement (*n* = 23)

This subtheme addresses the pros and cons of various logistical elements, such as the group size and platform of engagement in impacting the overall experience of the participants. Changes in government policies due to the COVID-19 pandemic required participants to attend the program online or a hybrid version of the program. This provided a unique opportunity to understand the participant’s experience. When asked for their preference for an online or onsite experience after experiencing both, their responses were mixed. Some participants “*preferred the sessions to be onsite. For the tours, we could have a 360-degree view and could even whisper or share suggestions with our fellow group members.*… *Being onsite, it felt like we were in a class interacting with teachers and the group which gave us more inspiration and ambition to help others, and sometimes steal ideas (AHS081, 65-year-old, female).”* Others felt that “*there was no difference because it has been 2 years since the COVID-19 pandemic [at the time of the interview], and we have been doing everything online. The visits to the museum would be better held in-person, whereas doing the rest of the work at home gave us more time. We can have a coffee, lie down, and rest (AHS100, 68-year-old, male).”* Program facilitators noticed a difference in the behavior and level of engagement between the online and onsite program where participants “*were more open in the gallery space and*… *the value-add of the onsite experience for them was not only through the museum tours but being together in the gallery space so that they can share their opinions. Doing the artmaking together as a group was much more positive and enriching for them than doing the artwork at home individually (Docent, NGS02).”*

Participants then suggested a different hybrid approach which included having an onsite museum tour and online artmaking sessions. Overall, their responses suggested that engaging in the program online was convenient and comfortable, however, it lacked the opportunities for organic interactions and guidance from the facilitator which was easily accessible in the onsite experience as explained by this participant, “*the interactions were quite dynamic and interesting, because I could hear a few voices at the same time. Unlike zoom, [where the interactions were] one [voice] in [and] one [voice] out. Listening to a lot of voices sometimes helped me to understand what the other guy was thinking or others’ work in progress*… *it made me realize that it was possible to see things from another angle which was not possible when I was on zoom (AHS076, 70-year-old, male).”* This might be a better alternative as the facilitators suggested that the current hybrid sessions were not sustainable for them because the sessions required them to “*mentally juggle and see participants both on zoom and in-person (Researcher and co-facilitator, FAC01)*” and their “*energy was completed zapped because they could not focus on both the in-person or the online session*… *despite the co-facilitator’s help with the online session participants (Artist-facilitator, FAC03).”*

## 4 Discussion

The objective of this study was to understand the lived experience and impact of the Singapore A-Health Intervention on the health and wellbeing of community dwelling older adults. A qualitative approach was adopted to address the research objectives by eliciting lived experiences from the intervention group participants and triangulated with inputs from the session facilitators. The program evaluation survey and acceptability focus groups revealed that participants were overall satisfied with the program implementation. The analysis identified interrelated themes on the intervention design, wellbeing outcomes, and enabling factors which supported the successful execution of the Singapore A-Health intervention. The outcomes of improved interpersonal bonds, personal growth, and mindful living were consistent with the literature on art and museum-based interventions for older adults (23, 24). Population based studies showed the positive associations of active participation in arts and cultural activities with life satisfaction, perceived health, quality of life, spiritual wellbeing, and reduction in depressive symptoms (25–27). As observed by one of the facilitators, the experience of a flow state during the intervention which is the complete absorption and enjoyment in an activity could explain the sense of mastery, satisfaction, and fulfilment reported by the participants (28). Furthermore, the participant’s reports of personal growth, autonomy, and self-acceptance were documented dimensions of Ryff’s six-dimensional model of psychological wellbeing (29). The social element of the program through group discussions and weekly dialog with the facilitators fostered a sense of belonging and support among participants, thereby strengthening social wellbeing. This newly formed group membership could also shape an individual’s self-concept which has a positive impact on self-esteem, as explained by the social identity theory (30, 31). Guided engagement with the artworks also encouraged participants to be present in the moment and fully immerse themselves in the creative process (32) which is beneficial to multiple areas of the older adults’ lives (33). These elements of immersion, socialization, and reflectiveness integral to the Singapore A-Health intervention experience were also key mechanisms posited in Tay and colleague’s conceptual framework for the role of arts and humanities in human flourishing (34). These positive outcomes are known factors that influence resilience and motivation among older adults, which was associated with increased medical adherence and health behaviors (35).

In addition to the impact of the program, this investigation revealed the complexities apart from the art modality that could influence the overall experience and the outcome of the intervention which is rarely documented in the literature. For instance, it was found that the intervention was intellectually stimulating and elicited positive stress among participants as well as the motivation to seek peer support. Yet, the same peer interaction during the program could be both a source of stressor and strength among the participants. There is a risk of psychological harm if these complexities are not adequately managed, and the current findings suggested possible enabling factors for successful implementation of art-based activities for health in the museum space. One key enabler is through facilitation, which comprised of communication, empathy, and flexibility. Clear and timely communication was essential, starting from the recruitment process where information to participants were clearly disseminated and concerns adequately addressed. During the program, the docents created a welcoming space at the museum while setting the context for the art facilitator to establish program structure and deliver the art program. Therefore, for multi-facilitator programs such the Singapore A-Health Intervention, it is of great importance for facilitators to have a strong understanding of the program (e.g., program aims, intended outcomes, session design). Moreover, the importance of exercising attentiveness (36) and empathetic communication by the facilitators creates a safe space for growth and learning (37, 38). This sets a supportive, non-judgmental culture for healthy competition and collaboration amongst participants. The findings from the focus groups also showcased diverse views from the participants regarding the intervention and support provisions, underscoring that a one-size-fit-all program is simply a mirage. This highlights the challenges of implementing art programs to a group with diverse preferences which may require more flexibility and adaptation on the facilitators’ end. For future practice, facilitators could adopt the person-centered art practice with communities framework to attend to the social, personal, cognitive and cultural dimensions of the participants in program implementation (39).

Another key enabler is the program design, where a culturally appropriate and structured curriculum is crucial. While the A-Health framework was based on rich empirical evidence, the 12-week Singapore A-Health Intervention activities were co-created with the NTU research team together with the Gallery’s community access team to curate artworks that were accessible to the older population in Singapore. These activities were further refined by the participants’ input for a better fit between the program and the target population. In practice, designing art-based programs with a participatory action research approach (15) aids in successful implementation and minimizes attrition rates in the community. Moreover, this study supports the use of a structured curriculum with deliverables and achievable milestones. The milestones enabled participants to evaluate their progress, and achieving these goals with a tangible indicator (i.e., art pieces) was rewarding for the participants. When communicated clearly to participants, this structure was particularly helpful for those who joined the program with little to no knowledge of art. In addition, the inclusion of monthly themes created further constraints which has been proven to facilitate the creative process (40). Taken together, insights from this study suggested that a structured and well-facilitated creative intervention could play a role in the enhancement of health and wellbeing at old age.

### 4.1 Limitations and future directions

While the study yielded valuable insights into the overall experience and impact of the Singapore A-Health Intervention, there are several limitations that should be considered. Regarding the sample composition, majority of the participants were female, of Chinese ethnicity, possessed a college degree, were retirees. Based on the recruitment criteria, only participants who were fluent in English and had access to the internet were included. This study therefore has moderatum generalizability to similar profiles of older adults. Future studies could translate the Singapore A-Health Intervention into Malay, Tamil, and Mandarin Chinese to increase access and test its applicability to other populations of older adults in Singapore. Moreover, participation in the focus group for this study was on an opt-in basis, which may result in a group self-selected profile of participants that is highly motivated and biased toward the program. Potential bias has been mitigated in this study through the careful crafting of the semi-structured topic guide (e.g., neutrally worded open-ended questions), neutral facilitation (e.g., encouraging open dialog among participants and encourage diversity in views), member checking (e.g., discussing preliminary insights with participants and allowing them to provide clarification on their experiences), and triangulation. As the qualitative responses of the participants are equally important as the quantitative data in providing a holistic understanding the impact of art and museum-based intervention, future research could consider incorporating the qualitative evaluation as an integral part of the investigation to include a wider range of participants. Participants could voluntarily opt-out of the focus group and withdraw their participation at any time instead of an opt-in basis. Finally, the synergy and collaboration among stakeholders in driving successful implementation were highlighted in the findings and warrants further investigation to inform practice and sustainability in the field.

## 5 Conclusion

The qualitative investigation of the Singapore A-Health intervention provided deeper insights to the improvements in wellbeing among older adults in Singapore, complementing the quantitative findings. Furthermore, enabling factors and complexities which drive successful implementation were uncovered. These findings provide a culturally unique perspective on the benefits of art and museum interventions and showcases the strengths of cross-industry collaborations in supporting the health and wellbeing of the ageing population.

## Data availability statement

The datasets presented in this article are not readily available because the original contributions presented in the study are included in the article/supplementary material, further inquiries can be directed to the corresponding author. Requests to access the datasets should be directed to AH, andyhyho@ntu.edu.sg.

## Ethics statement

The studies involving humans were approved by NTU Institutional Review Board (NTU-IRB). The studies were conducted in accordance with the local legislation and institutional requirements. The participants provided their written informed consent to participate in this study.

## Author contributions

AH and OB designed the study and obtained funding. MT designed the art and museum engagement activities. SM, SG, GY, AT, YY, and KG involved in the implementation of the research study. AH, MT, and SM conducted the analysis. All authors contributed to data interpretation, as well as the writing and revision of the manuscript.
